# Clinicopathological characteristics and outcomes of malignant adenomyoepithelioma of the breast: a single institution’s experience

**DOI:** 10.1186/s12957-022-02593-3

**Published:** 2022-04-22

**Authors:** Heba Mohammad Abdulla Alqudaihi, Sae Byul Lee, Byung Ho Son, Sei-Hyun Ahn, Jong Won Lee, Beom Seok Ko, Hee Jeong Kim, Il Yong Chung, Jisun Kim, Gyungyub Gong

**Affiliations:** 1 Department of Breast Surgery, Asan Medical Center, Collage of Medicine, University of Ulsan, Seoul, Republic of Korea; 2 Department of General Surgery, Qatif Central Hospital, Ministry of Health, Qatif, Eastern Province Saudi Arabia; 3 Department of Pathology, Asan Medical Center, Collage of Medicine, University of Ulsan, Seoul, Republic of Korea

**Keywords:** Breast, Malignant adenomyoepithelioma, Estrogen receptor, Subtype, Metastasis

## Abstract

**Background:**

Malignant adenomyoepithelioma of the breast is a rare tumor and most of relevant literature consists of individual case reports. This study objective was designed to evaluate clinicopathological features and treatment outcomes of 15 cases of malignant adenomyoepithelioma at a single institute.

**Methods:**

A retrospective medical record review was performed for 15 subjects confirmed with malignant adenomyoepithelioma upon postoperative pathological diagnosis at the Asan Medical Center from January 2008 to June 2018. Data regarding age at diagnosis, preoperative biopsy results, operation methods, the status of hormone receptors and HER2, and clinical outcomes were collected.

**Results:**

All cases were female patients diagnosed at median age of 50 years. Preoperative core needle biopsy results showed that 40% of the cases (6 out of 15) were benign which was in discordance with the final malignant pathology report. Thirteen cases underwent wide excision with or without sentinel lymph node biopsy (SLNB) and 2 cases had total mastectomy with SLNB. Five of 11 cases (45.5%) were triple negative. Ten of 15 cases underwent postoperative radiation therapy, 3 cases underwent chemotherapy, and 5 cases underwent endocrine therapy. During median follow-up of 55 months, the 5-year overall survival rate was 87.5% and the 5-year disease free survival rate was 91.7%. Two lung metastases developed. One case showed local recurrence 3 years after surgery and radiotherapy and subsequently developed lung metastasis 1 year late. Another case developed lung metastasis one and a half years after surgery in combination with endocrine therapy and neoadjuvant chemotherapy.

**Conclusion:**

Preoperative core needle biopsy showed inaccurate results for diagnosing malignant adenomyoepithelioma. Malignant adenomyoepithelioma has a high rate of triple negative subtype but has a relatively good prognosis although there is a risk of local and systemic recurrence.

## Introduction

Malignant adenomyoepithelioma or adenomyoepithelial carcinoma of the breast is a rare tumor, for which only a limited number of reports have been published. It was first described by Hampel in 1970 and further classified by Tavassoli in 1991 [1, 2]. Many adenomyoepitheliomas have demonstrated benign behavior and are often cured with excision with negative margins, but some have exhibited malignant transformation of the myoepithelial cells, ductal epithelial cells, or both. When one of these two components is histologically malignant, it is termed malignant adenomyoepithelioma [3]. Easily recognized malignant features include irregular invasive margins with surrounding stromal reaction, cellular atypia and pleomorphism, necrosis, and a high mitotic count throughout the tumor. Overgrowth of myoepithelial cells, high cellularity, and satellite foci are also considered features of malignancy [4–6]. Most malignant adenomyoepithelioma are cured with wide excision with negative margins, but some may demonstrate local recurrence or metastasis. However, since this disease is not common, clinical features, imaging findings, histopathological findings, clinical progress, and prognosis are not clearly defined or well-organized and are mostly reported as literature reviews of individual case reports.

This study was conducted to evaluate clinicopathological features and treatment outcomes of malignant adenomyoepithelioma by analyzing 15 patients with malignant adenomyoepithelioma of the breast treated in a single institute.

## Methods

A retrospective review of medical records of 19 cases of malignant adenomyoepithelioma in the database of the Asan Medical Center from January 2008 to June 2018 was performed. Four out of 19 cases were excluded. A specialized breast cancer pathologist reviewed pathology slides of all cases and excluded an invasive ductal carcinoma case and a case of concomitant invasive ductal carcinoma with malignant adenomyoepithelioma after the final postoperative pathology. Two cases were treated in another institute. The remaining 15 cases were reviewed by the same pathologist who confirmed the diagnosis of malignant adenomyoepithelioma. Clinical information included preoperative biopsy report, age at diagnosis, method of surgery, hormone receptor and HER2 status, adjuvant treatment, and clinical outcome whether or not recurrence occurred.

## Results

All 15 cases were women and age at diagnosis ranged from 34 to 67 years (median of 50 years). Except for one case, all other cases were utilized core needle biopsies (CNB) for preoperative histological diagnosis of tumors. Histologically, 12 cases showed benign to suspicious initial diagnosis varying between adenomyoepithelioma and papillary features. The preoperative results showed that 40% of the cases (6 out of 15) were benign which was in discordance with the final malignant pathology findings. Thirteen cases underwent wide excision with or without SLNB and 2 cases had total mastectomy with SLNB. No lymph node metastasis was found in all cases that underwent SLNB. For adjuvant treatment, 10 cases (66.7%) underwent radiation therapy, 3 cases (20%) underwent chemotherapy, and 5 cases (33.7%) underwent endocrine therapy (Tables 1 and 2). Mitosis in the tumor ranged from 3 per 10 HPF to 5 per 10 HPF (in 6 cases mitosis count was not identified). Microcalcification was seen in 4 cases and was not identified in two cases. Pathologic tumor sizes ranged from 4 mm to 70 mm. All cases showed clear resection margin of 1 mm or more. Five cases out of 11 cases were estrogen receptor positive and other 6 cases were negative. Only one case showed HER2 positive in immunohistochemical staining. Five of 11 cases (45.5%) were triple negative. All cases had no lymphovascular invasion except two cases, which were not identified (Table 3). During median follow-up of 55 months (5~162 months) after surgery, the 5-year overall survival rate was 87.5%, and the 5-year disease free survival rate was 91.7%. Two out of 15 cases had recurrence. One case (case no. 12) developed local recurrence with a disease-free interval of 40 months and lung metastasis. The other case (case no. 15) had lung metastasis.

**Table 1:** Demographic and clinical characteristics for all patients

Characteristics	All patients *n* = 15 (%)
Age (years)	
≤ 40	4 (26.7)
41–50	3 (20)
51–60	3 (20)
≥ 60	5 (33.3)
Surgery	
Breast conserving surgery	13 (86.7)
Mastectomy	2 (13.3)
Radiotherapy	
Yes	10 (66.7)
No	5 (33.3)
Chemotherapy	
Yes	3 (20)
No	12 (80)
Endocrine therapy	
Yes	5 (33.3)
No	10 (66.7)
SLNB	
Yes	6 (40)
No	9 (60)
Estrogen receptor status	
Positive	5 (45.5)
Negative	6 (54.5)
Not recorded	4
HER2 status	
Positive	1 (10)
Negative	9 (90)
Not recorded	5
Tumor size (mm)	
0–10	5 (33.3)
11–20	7 (46.7)
> 20	3 (20)
Mitosis count/10 HPF	
0–2	0
2–4	8 (53.3)
> 4	1 (6.7)
Not recorded	6 (40)

*SLNB* Sentinel lymph node biopsy

**Table 2:** Summary of all 15 cases

Case	Result of preoperative CNB	Final pathology	Method of surgery	Chemotherapy	Radiotherapy	Endocrine therapy	Follow-up (months)	Recurrence
1	IDP	AME carcinoma	WLE	−	+	Tamoxifen	105	−
2	IDC	AME carcinoma with DCIS	BCS, SLNB	−	+		105	−
3	AME tumor	AME carcinoma	TM, SLNB	Adjuvant AC 4 cycles	−		72	−
4	AME carcinoma	AME carcinoma	WLE, SLNB	Adjuvant TAC 6 cycles	+		37	−
5	AME neoplasm	AME carcinoma	WLE	−	+		33	−
6	Eccrine spiradenoma	AME carcinoma	WLE	−	−		20	−
7	Papillary neoplasm	AME carcinoma	WLE, SLNB	−	+	Tamoxifen	14	−
8	AME carcinoma	AME carcinoma	WLE	−	+	Tamoxifen	8	−
9	IDC	AME carcinoma	BCS, SLNB	−	+		54	−
10	Papillary neoplasm	AME carcinoma	WLE	−	+	Tamoxifen	45	−
11	AME neoplasm	AME carcinoma	WLE	−	+		33	−
12	AME neoplasm	AME carcinoma	WLE	−	+		162	Local recurrence, lung metastasis
13	Sclerosing adenosis	AME carcinoma	WLE	−	−		111	−
14	Papillary neoplasm	AME carcinoma	WLE	−	−		5	−
15	IDC	AME carcinoma	TM, SLNB	Neoadjuvant AC 4 cycles	−	Tamoxifen	24	Lung metastasis

*AME* Adenomyoepithelial, *IDP* Intraductal papilloma, *IDC* Invasive ductal carcinoma, *WLE* Wide local excision, *BCS* Breast conserving surgery, *SLNB* Sentinel lymph node biopsy, *TM* Total mastectomy, *AC* Adriamycin, cyclophosphamide, *TAC* Docetaxel (Taxotere), adriamycin, cyclophosphamide

**Table 3:** Histological characteristics of all 15 cases

Case	Tumor size (mm)	IHC (ER, PR, HER2)	Ki-67%	LVI	Clear RM (mm)	Mitosis count/HPF	Microcalcification
1	13	(+/+/−)	NA	−	1	4/10	+
2	15	(−/−/−)	10−20	−	1	4/10	NA
3	25	(−/−/−)	30	−	> 10	NA	−
4	16	(−/−/−)	30–40	−	3	4/10	−
5	10	NA	NA	−	1	3/10	−
6	28	(−/−/−)	20–30	−	1	5/10	−
7	8	(+/+/−)	< 10	−	2	4/10	−
8	15	(+/+/−)	20–30	−	5	NA	−
9	4	(−/−/+)	10–20	−	1	NA	−
10	13	(+/−/−)	10–20	−	5	4/10	−
11	10	NA	NA	−	1	3/10	−
12	70	(+/+/NA)	NA	−	1	NA	NA
13	16	NA	NA	NA	NA	NA	+
14	8	NA	NA	NA	NA	3/10	+
15	17	(−/−/−)^a^	30–40	−	9	NA	+

*NA* Not available, *IHC* Immunohistochemistry, *LVI* Lymphovascular invasion, *RM* Resection margin

^a^ER/PR weak positive in the preoperative CNB tissue

### Recurrence case presentations

#### Case 1 (no. 12)

**Fig. 1:**
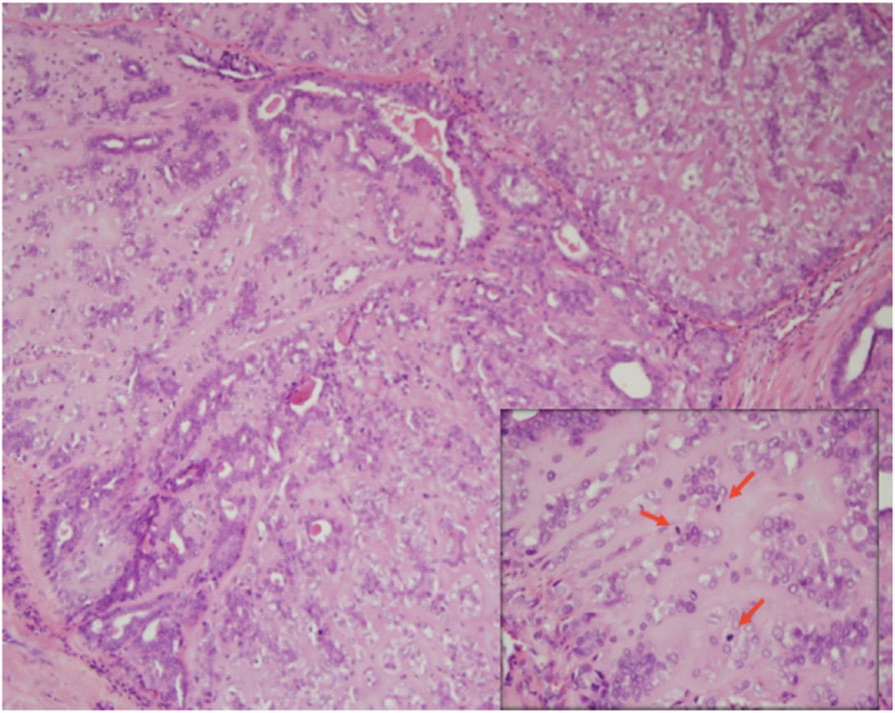
Microscopic finding of the breast tumor shows irregular nodularity with nests of atypical myoepithelial cells. Red arrows indicate high mitotic count (H&E stain)

**Fig. 2:**
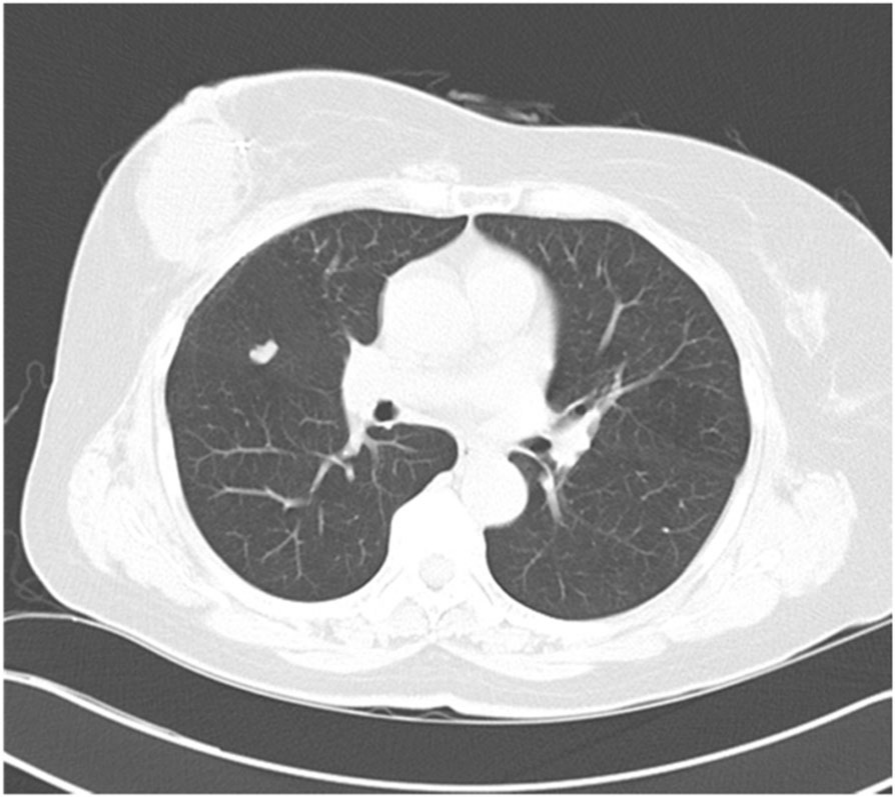
Chest CT shows a 1-cm sized, lobulating contoured nodule in the right middle lobe, attaching to the minor fissure

**Fig. 3:**
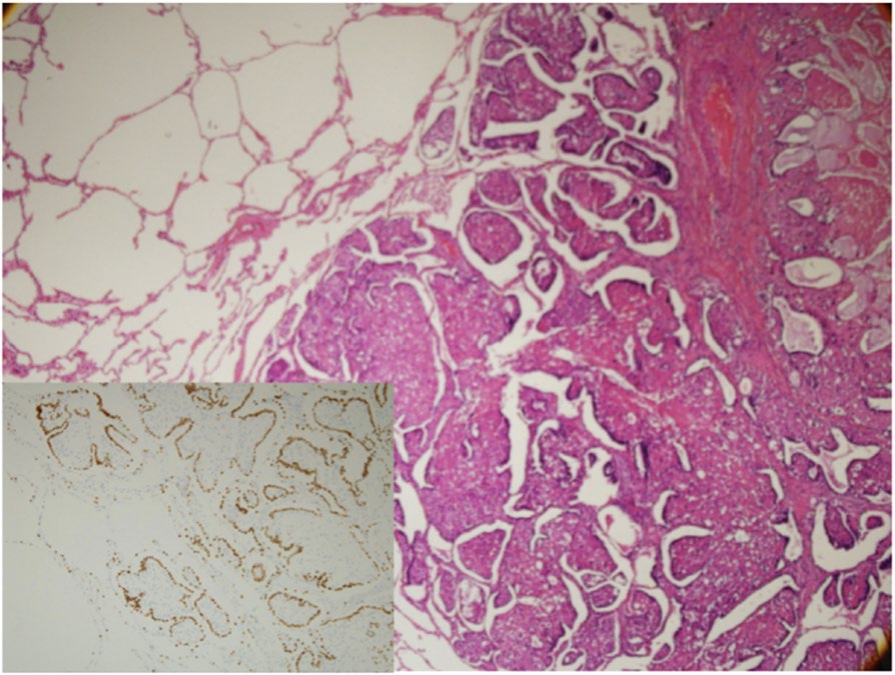
Microscopic finding of the lung metastasis shows interstitial growth pattern with entrapment of proliferating bronchoalveolar epithelium between normal lung tissue (H&E stain). Small picture shows lung epithelium lining (TTF stain)

A 65-year old woman presented with right breast mass and CNB showed uncertain malignancy of an adenomyoepithelial tumor. Wide excision was done. Final histology showed myoepithelial carcinoma with < 1 mm resection margins (Fig. 1). Immunohistochemical staining for receptors showed triple negative markers. The patient received adjuvant radiotherapy. After postoperative follow-up of 40 months, she developed local recurrence and underwent wide excision once more. Metastatic nodule at the middle lobe of right lung developed and was seen on chest CT 1 year later and treated with wedge excision and adjuvant tamoxifen (Figs. 2 and 3). Clinical follow up for 10 years after metastasis management was uneventful.

#### Case 2 (no. 15)

**Fig. 4:**
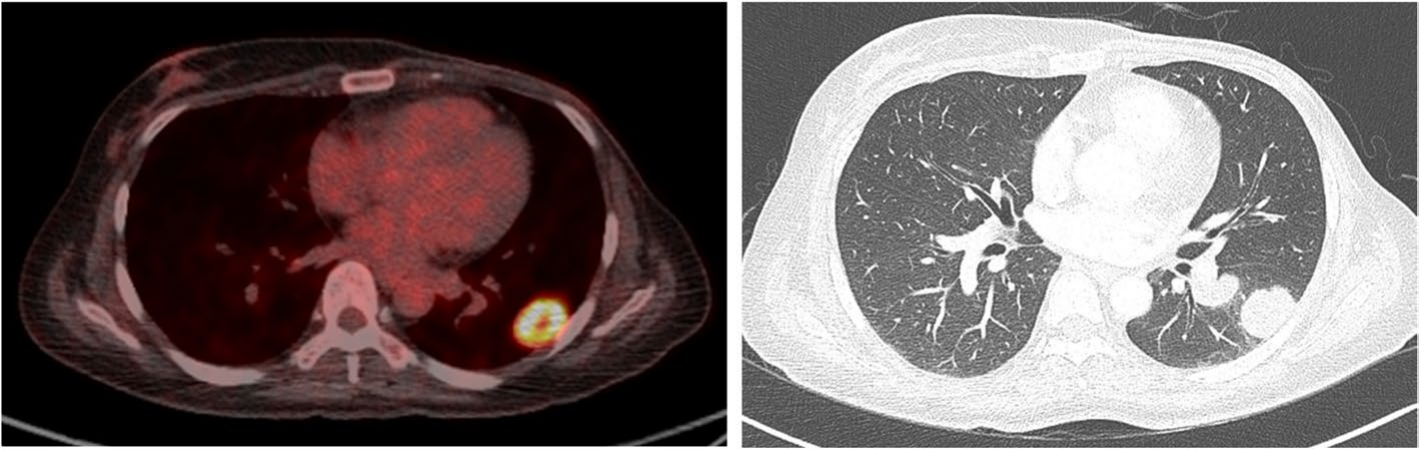
Both PET and chest CT showed multiple metastases in the left lung at one and a half years after surgery

A 34-year old woman presented with a left breast mass. Preoperative CNB showed invasive ductal carcinoma. Total mastectomy with SLNB was done after receiving neoadjuvant chemotherapy (AC 4 cycles). Final pathology results showed malignant adenomyoepithelioma with 9 mm free resection margins. Immunohistochemical staining for receptors in the tumor showed triple negative. She received adjuvant tamoxifen therapy according to the result that both estrogen and progesterone receptors showed weak positive in the initial CNB tissue. She developed lung metastasis at one and a half years after surgery in clinical follow-up (Fig. 4). Two years later, she died of disease progression without response to chemotherapy.

## Discussion

Although our retrospective clinical study sample size of malignant adenomyoepithelioma was small, the results showed that preoperative core needle biopsy results might be inaccurate for malignant pathology, and the disease has a high rate of triple negative subtype, and a risk of local recurrence as well as lung metastasis.

The diagnosis of malignant adenomyoepithelioma on a needle core biopsy or intraoperative frozen section biopsy can be difficult because of pathologically morphologic heterogeneity. In limited biopsy material, the sampled tissue may even be mistaken for invasive carcinoma especially in tumors that have compact glandular structures with clear cell epithelioid myoepithelial proliferation [7–11]. Our study showed that 40% of the cases were benign on preoperative core needle biopsy contrary to the final malignant pathology and supports for the limitation in the use of core needle biopsy for pathological diagnosis of malignant adenomyoepithelioma. Preoperative discordant diagnoses of core needle biopsy were papillary lesions like papillary neoplasm and intraductal papilloma, eccrine spiradenoma, sclerosing adenosis, benign adenomyoepithelioma, and invasive ductal carcinoma. Wiens [11] reported a significant discordance between initial and final diagnosis of adenomyoepithelioma in that 50% of samples previously diagnosed as adenomyoepithelioma or adenomyoepithelial lesions were reclassified as myofibroblastoma, sclerosing papilloma, radial scar, and fibroadenoma with myoid metaplasia. Because atypical features such as pronounced nuclear pleomorphism, mitotic activity, necrosis, invasive growth, and the overgrowth of 1 of the 2 components of the lesion may not be evident in the needle core biopsy, excisional biopsy is recommended to rule out a carcinoma arising in an adenomyoepithelioma. In addition, immunohistochemical staining for myoepithelial markers, especially p63, could be useful for highlighting the abundant myoepithelial components [8]. A recent study suggested three variants of malignant adenomyoepithelioma (M-AME): M-AME in situ, M-AME invasive, and AME with invasive carcinoma [12]. In our study, we excluded a case of concomitant invasive ductal carcinoma with malignant adenomyoepithelioma on the postoperative final pathology because invasive ductal carcinoma is considered a more significant form of malignant disease and its treatment is different from that of malignant adenomyoepithelioma.

Although there are not many studies determining ER/PR and HER2 data in malignant adenomyoepitheliomas, the tumors may express the phenotypic features of the basal-like breast carcinoma [10, 13, 14]. In our study, 45.5% of the tumors showed triple negative subtype, and 54.5% were estrogen receptor negative. The rate of triple-negative subtype in malignant adenomyoepithelioma seems to be much higher than the rate of triple-negative subtype expressed in invasive ductal carcinoma. Salivary gland-type tumors of the breast likely present the unusual feature of usually exhibiting a triple negative phenotype but with low-grade behavior in contrast to triple-negative tumors of the breast which have a worse prognosis [15].

There were one local recurrence and two lung metastases in our study. One case developed local recurrence followed by lung metastasis. Local recurrence might be related to performing a narrow surgical resection margin in the initial surgery. Wide complete excision of malignant adenomyoepithelioma is recommended for adequate treatment as acquiring a wide negative surgical margin is an important strategy for preventing local recurrence. Although there is a little objective evidence to support a role for radiotherapy or chemotherapy in the management, postoperative radiotherapy might be applied for patients with high risk to lower the recurrence risk. 66.7% of patients in our study underwent radiotherapy. If a lesion recurs, a repeat wider excision or mastectomy should be required. If mastectomy is planned for malignant adenomyoepitheliomas, sentinel node biopsy may be indicated for axillary evaluation. However, axillary lymph node metastasis was very rare [2, 9, 10]. There was no metastasis in four SNB cases in our study. The possibility of direct extension of the tumor to the lymph node might be taken into consideration in the case of proximity of the node to the primary lesion.

Hematogenous metastasis of malignant adenomyoepithelioma is common including lung, liver, brain, bone, and thyroid metastases, and the prognosis of patients is extremely poor with the metastasis [4, 16–18]. One third of patients suffered from distant metastases and local recurrence which tended to occur 4 months to 2~3 years after the first diagnosis [17]. Our study also supported the finding for the risk of metastasis of malignant adenomyoepithelioma. A recent study from the NCDB reported 5-year OS of 74.4% during 52 months of median follow-up [19]. In this study, the 5-year OS was as high as 87.5% during median follow-up of 55 months. More radiation therapy and endocrine therapy were performed in the study. In both studies, chemotherapy was performed at a low rate of only about 20%, which is thought to be due to the lack of evidence for the benefit of chemotherapy in malignant adenomyoepithelioma. Not enough is understood about clinical benefit of endocrine therapy for malignant adenomyoepithelioma because of a lack of data thus far. Because estrogen receptor tends to be negative, endocrine therapy might be not indicated. However, in our study, half the cases had positive estrogen receptor underwent endocrine therapy. More studies are needed to evaluate the clinical benefits of endocrine therapy.

There are several limitations in our study. Because this study involved a small sample-sized retrospective clinical series, results could not be shown as statistically driven data, and there were no comparison studies. We did not acquire genomic expression data from primary or recurrence tumors. These genomic data might extend our understanding about the tumor biology and metastasis of malignant adenomyoepithelioma in the future.

## Conclusion

Although malignant adenomyoepithelioma of the breast is an uncommon tumor, because preoperative core needle biopsy results showed a high discordant rate with the final malignant pathology, a careful pathological and radiological evaluation should be considered for acquiring an accurate diagnosis. Because malignant adenomyoepithelioma has a high rate of triple negative subtype and a risk of local recurrence as well as lung metastasis, wide complete excision of the tumor with a wide negative surgical margin is recommended for preventing local recurrence. There is a little objective evidence thus for to support a role for radiotherapy or chemotherapy. However, postoperative radiotherapy as well as endocrine therapy for patients with positive estrogen receptors might be applied for patients with high risk of recurrence.

## Data Availability

The data generated and/or analyzed in the current study are publicly available.

## Abbreviations

AME: Adenomyoepithelial
IDP: Intraductal papilloma
IDC: Invasive ductal carcinoma
WLE: Wide local excision
BCS: Breast conserving surgery
SLNB: Sentinel lymph node biopsy
TM: Total mastectomy
AC: Adriamycin, cyclophosphamide
TAC: Docetaxel (Taxotere), adriamycin, cyclophosphamide
NA: Not available
IHC: Immunohistochemistry
LVI: Lymphovascular invasion
RM: Resection margin
